# Are Global Breast Cancer Incidence and Mortality Patterns Related to Country-Specific Economic Development and Prevention Strategies?

**DOI:** 10.1200/JGO.17.00207

**Published:** 2018-06-08

**Authors:** Martine Bellanger, Nur Zeinomar, Parisa Tehranifar, Mary Beth Terry

**Affiliations:** **Martine Bellanger**, Ecole des Hautes Etudes en Sante Publique - University Sorbonne Paris Cite, Paris, France; **Nur Zeinomar**, **Prisa Tehranifar**, and **Mary Beth Terry**, Columbia University; **Parisa Tehranifar** and **Mary Beth Terry**, Herbert Irving Comprehensive Cancer Center, Columbia University Medical Center, New York, NY; and **Martine Bellanger**, **Nur Zeinomar**, **Parisa Tehranifar**, and **Mary Beth Terry**, International Breast Cancer and Nutrition Project, Lafayette, IN.

## Abstract

**Purpose:**

There remains considerable international variation in breast cancer incidence and mortality, but a comprehensive examination of rates by country level economic, development and cancer prevention policies is lacking.

**Materials and Methods:**

We compared GLOBOCAN 2012 age-specific breast cancer incidence and mortality rates for 177 countries by using development and policy data available from the WHO Global Cancer Country Profiles data base. We classified each country on the basis of gross national income per capita from the World Development Indicators data base, as follows: low-income country (LIC), lower-middle–income country (LMIC), upper-middle–income country (UMIC), and high-income country (HIC).

**Results:**

There were 1,651,326 breast cancer cases and 516,868 breast cancer deaths estimated in 2012. Approximately three quarters of all breast cancer cases and 60% of the breast cancer deaths were in women from HICs and UMICs. Age and country-level income explained approximately 60% of the international variation in breast cancer incidence and mortality in women of all ages (adjusted *R*^2^ = 58% and 60%, respectively). Economic development indicators additionally increased the overall variation in incidence and mortality by approximately 5%. In women younger than age 50 years, country-level income explained 68% of incidence and 59% of mortality; economic development indicators additionally increased this percentage by approximately 4%. Country-level cancer prevention policy indicators contributed little to explanation of the overall variation in incidence and mortality after analysis accounted for age and country-level income; however, an overall resource summary index of greater economic development and cancer prevention policies was related to lower mortality within each major income level.

**Conclusion:**

Although breast cancer incidence increases with higher income levels in all ages, women in the poorest countries bear a relatively higher burden of breast cancer mortality, particularly women younger than age 50 years.

## INTRODUCTION

Breast cancer remains the most common type of cancer in women; there were an estimated 2.4 million incident cases and 523,000 deaths in 2015.^1^ Changes associated with global economic and sociodemographic trends, such as increasing population and aging populations^1^ and concomitant changes in lifestyles and environmental exposures, contribute to the cancer burden in high-income countries (HICs) as well as in low-income countries (LICs) and lower-middle–income countries (LMICs).^2,3^ As reported by the Global Burden of Disease Cancer Collaboration,^4^ although countries of all income levels experienced increases in breast cancer incidence, there is heterogeneity in disease burden across countries of different income levels, as estimated by the disability-adjusted life-year, a common metric that combines duration and quality of life and that can be applied across diseases and organs.^3^ Of note, approximately 69% of total disability-adjusted life years lost as a result of breast cancer were observed in LMICs.^1^

Breast cancer incidence rates are increasing in many LMICs, even if the absolute rates are lower in LMICs than in HICs.^3,5^ In addition, despite breast cancer mortality reductions in some HICs since 1990, other HICs with historically low mortality rates as well as many LMICs have experienced increased mortality rates.^3^ For example, although the United States had a steady decline in age-standardized breast cancer mortality rates between 1990 and 2013 (22.3 per 100,000 in 1990 to 13.4 per 100,000 in 2013), Japan’s breast cancer mortality rate increased from 6.3 per 100,000 to 9.1 in the same time period.^6^

Globally, trends in breast cancer in many LMICs are indicative of the so-called late phase of the epi transition and cancer transition—that is, the increase in breast cancer incidence, along with other cancers and noncommunicable diseases, in these countries reflects trends in wealthier countries and the associated Westernized lifestyles (ie, unhealthy diet, tobacco consumption, sedentary lifestyle) and reproductive patterns that confer a higher risk of breast cancer.^7,8^

Cancer prevention strategies, including secondary (screening) prevention, are not universally endorsed, implemented, or used.^2,3,5,8-10^ For example, controversies about the age of initiation and the frequency of mammography screening abound and have led to diverse country-specific screening policies, even among HIC countries.^2,3,5,11,12^The cost-effectiveness of mammography screening also has been questioned, because the amount of resources necessary to maintain national 1- to 2-year mammographic screening programs may be larger than the benefits of screening in some HICs.^5^ In LMICs and limited-resource settings, implementation of population-based mammographic screening program requires additional infrastructure, as recommended by WHO.^12,13^ In addition to variations in secondary prevention initiatives worldwide, tertiary prevention in the form of chemotherapy and radiation therapy can be effective, but the availability and uptake may be limited in LMICs and low-resource settings.^5,7,14-17^

Increasingly, countries also are engaging in primary prevention policies and guidelines, which include recommendations for chemotherapy prevention, such as tamoxifen and lifestyle modification. For example, the American Cancer Society recommends maintenance of moderate alcohol intake, healthy body weight, and regular physical activity—all of which have been associated with reduced breast cancer risk.^14,18^ Primary prevention through lifestyle modification affects other noncommunicable diseases,^18^ which yields benefits that extend beyond breast cancer.

It is unclear whether the contribution of economic development and prevention policy indicators can explain worldwide variation in breast cancer incidence and mortality. Here, we examine the extent to which age, country-specific income levels, development indicators, and cancer prevention policies and guidelines help explain the international variation in breast cancer incidence and mortality.

## MATERIALS AND METHODS

### Data Sources

We extracted age-specific breast cancer incidence and mortality data from GLOBOCAN 2012, produced by the International Agency for Research on Cancer, which provides country-level estimates of cancer incidence, mortality, and prevalence for 184 countries.^19^ Of these 184, World Bank indicators were available for 177 of these countries. We extracted gross national income (GNI) per capita in United States dollars (US$) by using the 2014 World Development Indicators (WDI) data base of the World Bank,^20^ and we classified these countries into income groups as follows: GNI per capita ≤ $1,045, LICs (n = 32); GNI per capita between $1,046 and ≤ $4,125, LMICs (n = 47); GNI per capita between $4,126 and ≤ $12,735, upper-middle–income countries (UMICs; n = 44); and GNI per capita > $12,735, HICs (n = 54).^20^

We extracted indicators of development from the WDI, including female life expectancy at birth and fertility rate in 2014, the percentage of women age 20 to 24 years who were first married by age 18 years in the period of 2009 to 2014, the percentage of women employed during the period from 2010 to 2014, women in parliament as a percentage of total seats in 2015, and the Gini index that measures inequality in country income distribution (from 0 for absolute equality to 100 for absolute inequality). We retrieved data on female education level as the percentage of women age 25 years or older who had at least secondary education during the period from 2005 to 2015, and we retrieved contraceptive prevalence in 2014 from the United Nation Development Program. In addition to two of the development indicators (fertility and oral contraceptive use), which are relevant to breast cancer incidence, we also incorporated county-level data about the percentage of children ever breastfed for any period of time, as reported by Victora et al.^21^

We used the WHO Global Cancer Country Profiles (from 2014) to access information about breast cancer prevention. This information included whether the country reported policies related to lifestyle risk factors (primary prevention), screening coverage with mammography (secondary prevention), and availability of radiotherapy and chemotherapy (tertiary prevention).

#### Statistical analysis.

After descriptive analyses, we used linear regression models to evaluate the extent to which incidence and mortality rates were explained by age, income, and the additional impact that country-specific development and cancer prevention policies had on explanations of differences in incidence and mortality. We examined the following independent variables: age, economic variables (eg, country income level and Gini index); female development indicators (percentage of women employed, percent of women with at least some secondary education, female life expectancy, fertility rate, contraceptive prevalence, and percentage of women ages 20 to 24 years who married at a young age); breastfeeding prevalence; and public prevention policies, including primary prevention (tobacco policy, alcohol control, physical activity–related programs), secondary prevention (screening, to include clinical breast examination and mammography), and tertiary prevention (access to cancer treatment).

In addition, we developed a country-level resource index to summarize the availability of resources in each country by summing the quartile rank of the seven WDI socioeconomic development indicators (with reverse coding ranks for certain indicators as appropriate, eg, fertility rate) and adding a value of 0 or 1 to represent the presence or absence, respectively, of each of the nine prevention policy indicators. The final score ranged from 4 to 37, and the highest score represented the countries with the highest rates of life expectancy, employment, education, contraceptive use, and cancer prevention policies as well as the lowest percentage of young women married, lowest fertility rate, and lowest Gini. We used this country-level resource index in descriptive analysis to capture the range and distribution of resources at the country level and in regression models stratified by country-level income. All statistical analyses were performed with SAS software version 9.4 (SAS Institute, Cary, NC).

## RESULTS

### Incidence and Mortality Patterns

**Fig 1:**
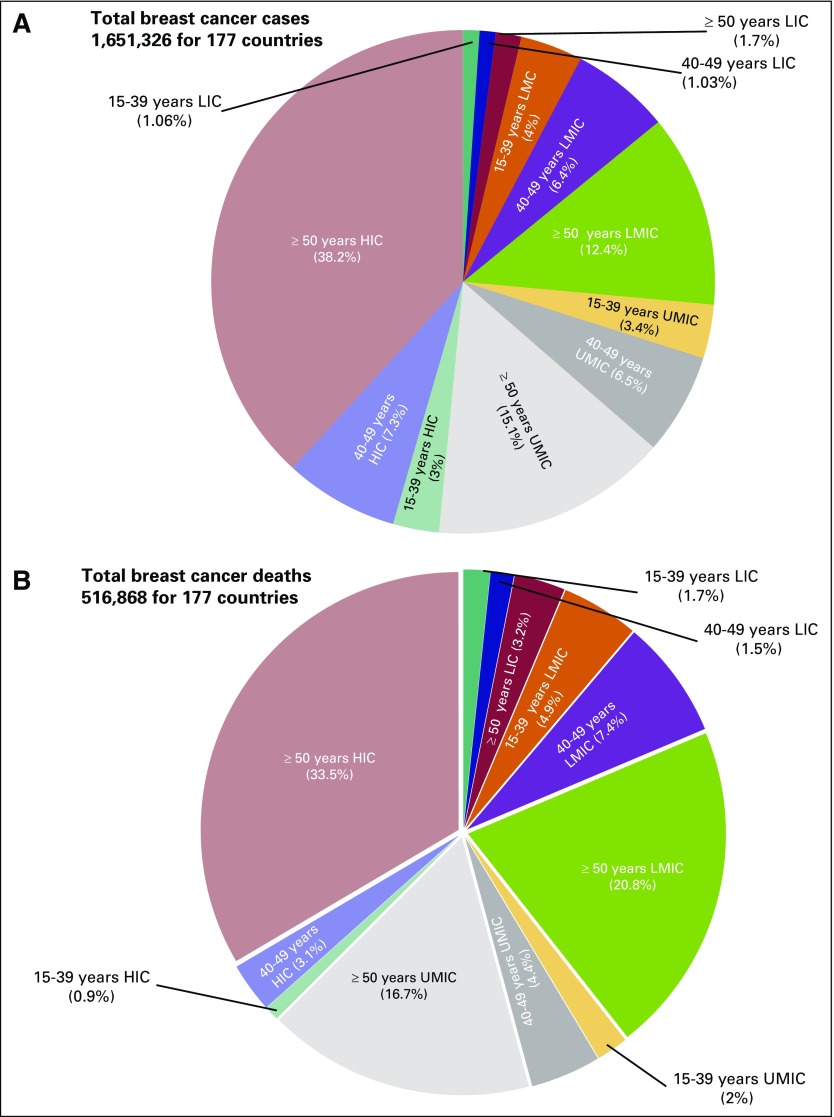
Breakdown of (A) incidence and (B) mortality by age and by country income level in 2012 according to GLOBOCAN International Agency for Research on Cancer (2012) & World Development Indicator (2014, 2015) data. LIC, low-income countries; LMIC, lower- to middle-income countries; UMIC, upper-middle–income countries; HIC, high-income countries.

In the 177 countries included in this study, there were an estimated 1,651,326 breast cancer cases and 516,868 breast cancer deaths in 2012. As shown in Figure 1, slightly less than three quarters of all breast cancer cases were in women from HICs and UMICs (48.5%, 25.1%, 22.2%, and 3.79%, for HICs, UMICs, LMICs, and LICs, respectively). The proportions of incidence in women younger than age 50 years were similar across the top three income levels (10.6%, 9.9%, 10.4%, and 2.8%, for HICs, UMICs, LMICs, and LICs, respectively). Sixty percent of the breast cancer deaths were in women from HICs and UMICs (37.5%, 23.1%, 33.1%, and 8.1%, for HICs, UMICs, LMICs, and LICs, respectively). The proportion of mortality in women younger than age 50 years was highest for LMICs (4%, 6.4%, 12.3%, and 3.2%, for HICs, UMICs, LMICs, and LICs, respectively).

**Fig 2:**
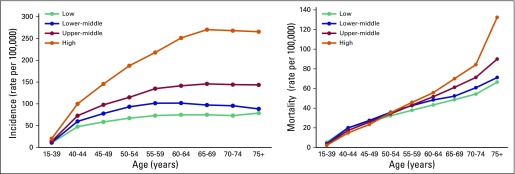
Average breast cancer incidence and mortality rates by age group and by country income level.

Figure 2 presents incidence and mortality rates by age group and income level. The highest incidence was found in the HICs across all age groups, and the slope of the age incidence curve flattened in the HICs at least 5 years later than in LICs. Differences in incidence rates between income levels were less pronounced among younger women than among women age 50 years and older (eg, the difference between HICs and LICs ranged from 9.8 to 114.3 per 100,000 in age groups younger than age 50 years and ranged from 120.3 to 195.2 per 100,000 in age groups older than age 50 years (Data Supplement). Mortality rates were higher in LICs and LMICs among women younger than age 50 years, and the largest differences were among women younger than age 40 years; with the highest average rate was in LICs, which was more than double that in HICs (5.4 *v* 2.4 per 100,000). Mortality rates for women in their 50s were similar across income groups but were slightly lower in LICs.

### Country-Level Development Indicators

Average female life expectancy at birth, percent of population with at least some secondary education, and percentage of women who used contraceptive increased with increasing country income levels. The percentage of women who were first married by age 18 years, the fertility rate, and the percentage of babies ever breastfed decreased by increasing country income level (Table 1). The percentage of women in parliament was similar across major income levels, as was the Gini.

**Table 1:**
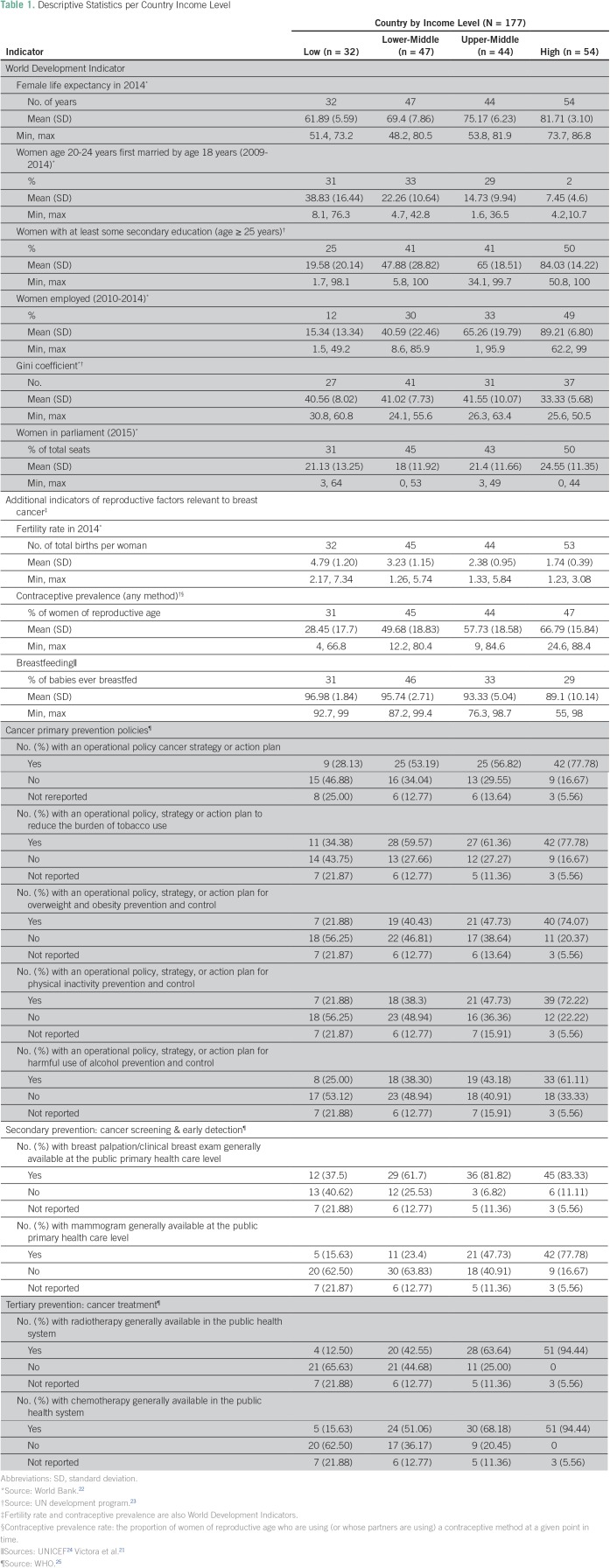
Descriptive Statistics per Country Income Level

### Cancer Prevention Policies

Cancer prevention strategies also increased with increasing income level (Table 1). Mammograms were available in less than 16% of LICs, in 23% of LMICs, in 48% of UMICs, and in 78% of HICs. Cancer treatments in the public health system, such as radiotherapy and chemotherapy, were available in 13% to 16% of LICs, 43% to 51% of LMICs, 64% to 68% of UMICs, and 94% of HICs. Compared with secondary and tertiary prevention strategies, primary prevention policies had less of an income gradient (Table 1).

### Multivariable Analysis of Factors Associated With Breast Cancer Incidence and Mortality Rates

Age group and country income level explained 58% of the overall variation in incidence and 68% of variations in women younger than age 50 years (Model 1; Table 2). Additional adjustment for economic development indicators (Model 2) increased the variations by 7% and 4%, respectively. Primary and secondary cancer prevention indicators (Model 3) contributed little to the explanation of the overall variation in incidence, although clinical breast exam and availability of mammography were negatively and positively associated with breast cancer incidence, respectively. Countries with high rates of breastfeeding also had lower incidences of breast cancer (*P* < .001). The Data Supplement provides additional examination of finer age stratifications for these incidence models.

**Table 2:**
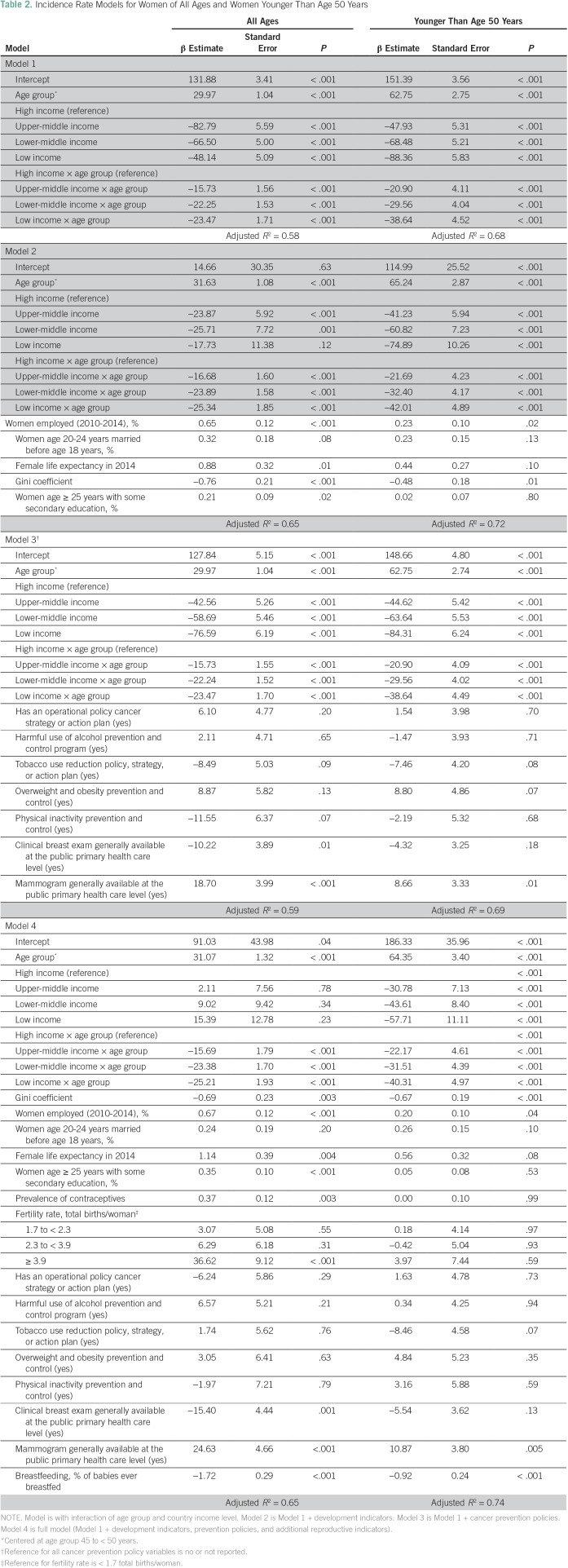
Incidence Rate Models for Women of All Ages and Women Younger Than Age 50 Years

Age and income groups explained 60% and 59% of mortality variation in women of all ages and in women younger than age 50 years, respectively (Table 3). In women younger than age 50 years, women from LICs and LMICs had higher mortality than women from HICs (*P* = .02 and *P* = .001, respectively). Additional adjustment for economic development indicators improved the variation in mortality rates in women of all ages and in women younger than age 50 years by 5% and 4%, respectively. Country-level cancer prevention policy indicators contributed little to explanations of the overall variation in mortality after analysis accounted for age and income, but the availability of radiotherapy was associated with lower overall mortality in women younger than age 50 years. The Data Supplement provides an examination of finer age stratification for these incidence models.

**Table 3:**
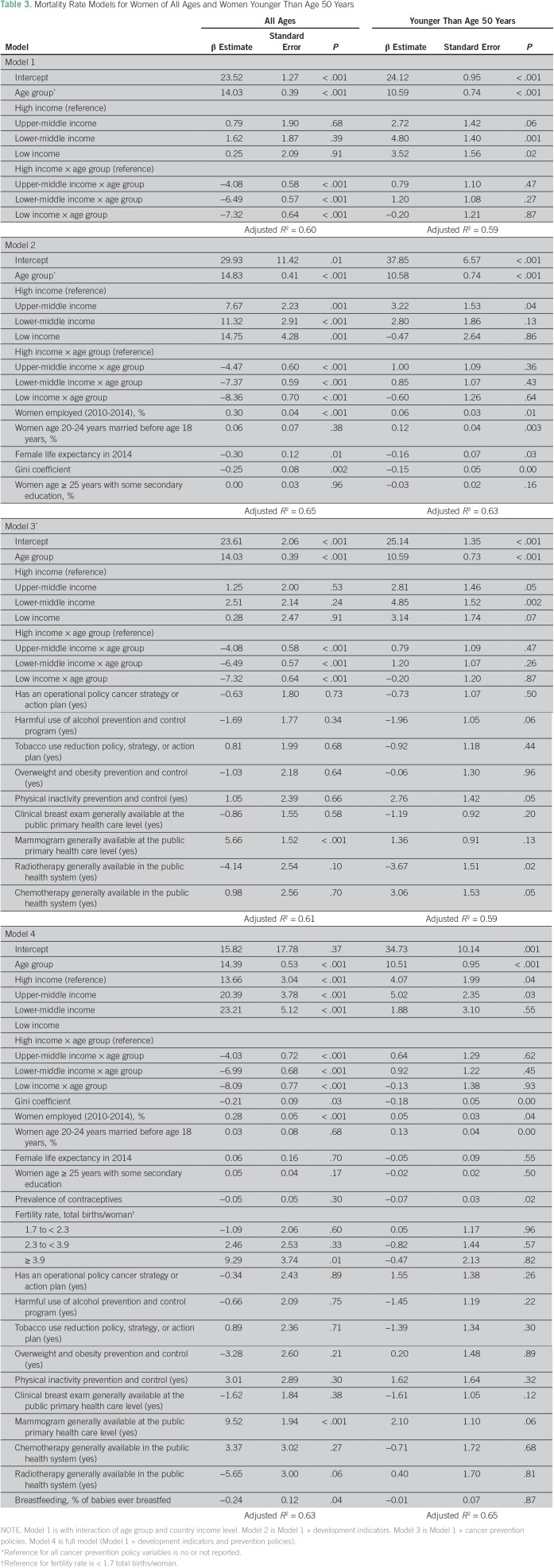
Mortality Rate Models for Women of All Ages and Women Younger Than Age 50 Years

### Country-Level Resource Index

**Fig 3:**
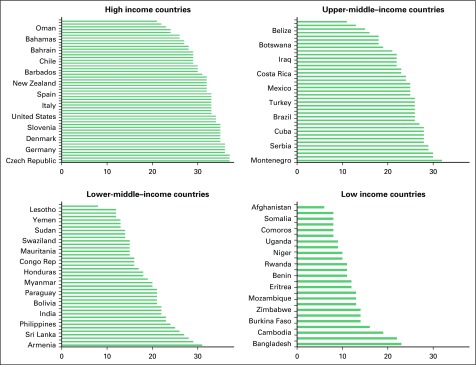
Countries ranked by country-level resource index in each income group level.

Figure 3 shows the variation by income in the summary resource index. The resources score ranged from 21 to 37 in HICs (median, 32), from 11 to 32 in UMICs (median, 25), from 8 to 31 in LMICs (median, 18), and from 6 to 23 in LICs (median, 11). There was some overlap in the resources scores between adjacent country income levels, but the score differed substantially between HICs and LICs; the lowest resource score in HICs was equivalent to the highest resource score in LICs (21 and 23, respectively). The Data Supplement provides information on average incidence and mortality rates by age group, which illustrates the heterogeneity in resources across age and income level by high and low incidence countries. The Spearman correlation coefficients were statistically significant (*P* < .001) between the summary resource index and both the incidence and the mortality rates, but the coefficient was much higher for incidence (0.47) than for mortality (0.08).

Country-level development and cancer prevention policies did not substantially alter the percentage variation explained in incidence and mortality after analysis accounted for age and income level. However, we examined whether the summary resource index could explain heterogeneity within major country income level. In models stratified by country-level income level, and after adjusting for age, this summary index level was positively associated with incidence but negatively associated with mortality; the summary index explained variation in models with women younger than age 50 models years, and this summary index also explained 73%, 69%, 52%, and 52% of the variation in mortality rates in women within HICs, UMICs, LMICs, and LICs, respectively.

## DISCUSSION

On the basis of data from 177 countries, we found that substantial variation in breast cancer incidence and mortality can be explained by age and country-level income. The country-level income differences in breast cancer incidence rates were smaller in women younger than age 50 years. Difference in incidence rates by income level after age 50 years were driven primarily by steeper age-related increases in incidence in women from HICs, as seen in previous studies.^14,26,27^ In women younger than age 50 years, mortality was high in women in LMICs and LICs compared with women in UMICs and HICs. In contrast, mortality rates for women older than age 50 years increased by increasing income levels, which followed the income pattern for incidence, although the income gradient for mortality was considerably smaller than that for incidence. These results suggest that, although breast cancer incidence increases with higher income levels, younger women in the poorest countries bear a relatively higher global burden of disease and years of life lost as a result of breast cancer mortality.^1^ Country-level development indicators additionally explained about 4% to 5% of incidence and mortality whereas cancer prevention strategies explained very little after analysis accounted for country-level income and age.

These findings highlight the central role of country-level income and economic development on increasing incidence of noncommunicable diseases such as breast cancer, and the findings are consistent with prior research.^5,8^ However, these results should not be interpreted as suggestions of inevitable or irreversible consequences of economic growth. Importantly, reductions in breast cancer mortality despite increasing incidence, as seen in HICs, may be possible in LICs but require additional research to ensure the successful and appropriate implementation in these settings. For example, even though economic development indicators and cancer policy strategies explained little of the variation after age and country-level income were considered, the summary index of development and cancer prevention policies was associated with lower cancer mortality within each major income level. To inform these efforts, our study considered and identified key prevention policies and development indicators that had significant associations with breast cancer incidence and mortality. For example, breastfeeding was associated with both lower incidence and lower mortality. Availability of country-level tobacco use reduction and physical inactivity prevention policies also were associated with lower incidence, albeit only of borderline statistical significance (*P* < .10). Primary prevention strategies were more likely than secondary and tertiary prevention strategies to be present in LICs. This confirms previous findings from the literature, in which primary prevention strategy was effective and cost-effective especially in LIC and LMIC setting, where the implementation of secondary strategies requires important resources.^7,17^ The health benefits gained from these interventions expand beyond breast cancer for noncommunicable diseases that have even stronger associations with tobacco use and obesity (eg, diabetes, cardiovascular diseases, lung and colon cancers) underscore the importance of such primary prevention as an economic investment.^7,17^

We recognize the limitation of extrapolating the findings on the basis of country-level data for interventions that have been effective at the individual level. For example, the associations of mammography with higher incidence without a reduction in mortality suggest an increased detection of asymptomatic and indolent breast cancers without mortality benefits from earlier stage of cancer, and this finding is consistent with prior research in this area.^2,3,5,11,12^ However the actual reported increase in mortality that we observed in women older than age 50 years suggests the fallacy of making inferences at the individual level according to country-wide data for individual-level interventions. For tertiary prevention strategies, available radiotherapy treatment also was associated with lower mortality rates, but chemotherapy was associated with higher morality in women younger than age 50 years.

Nevertheless, although inferences that are based on individual factors cannot be based on country-level data, the pattern of these development and cancer prevention policies can provide insights into the variability of global rates. Within each major income level, these policies also can explain a large amount of the variability; countries within each major income level differ substantially with regard to these indicators (Fig 3).

Another limitation is the cross-sectional nature of the study design, which did not allow evaluation of temporal trends in the rates or the independent variables examined. Changes in incidence and mortality rates are needed to evaluate changes in developmental and cancer prevention policies.

Country-level economic development as measured by income category helps explain approximately two thirds of the international variation in breast cancer incidence but explains a much smaller amount of breast cancer mortality, particularly in women younger than age 50 years. Notably, in women younger than age 50 years, mortality rates increased with decreasing country-level income—a pattern that differed from the stable-to-increasing mortality rates by increasing income in women older than age 50 years. Other indicators of economic development and country-wide primary and secondary prevention policies helped explain variation within major income level but not across major income levels. These results call for a greater attention to understanding the growing breast cancer incidence and mortality in younger women, for whom screening programs such as mammography are less likely to be used or successful to improve mortality.
